# Rapid molecular detection of TB drug resistance from swab samples: a proof of concept study

**DOI:** 10.5588/ijtldopen.26.0074

**Published:** 2026-07-13

**Authors:** T. Pfurtscheller, M.M. Golla, S. Jain, V. Dalay, S. Yerlikaya, D. Marcelo, A. Steadman, A. Pabruada, C.M. Denkinger, C. Yu, A. Gupta-Wright

**Affiliations:** 1 Department of Infectious Diseases and Tropical Medicine, University Hospital Heidelberg, Heidelberg, Germany;; 2 De La Salle Medical and Health Sciences Institute, Dasmariñas, Philippines;; 3 German Center for Infection Research DZIF, Partner Site Heidelberg, Heidelberg, Germany;; 4 Global Health Labs, Inc., Bellevue, WA, USA;; 5 Asian Hospital and Medical Center Manila, Muntinlupa, Philippines;; 6 Imperial College London, London, UK.

**Keywords:** tuberculosis, point-of-care molecular testing, non-sputum diagnostics, the Philippines

Dear Editor,

Diagnosis of TB remains largely dependent on sputum-based testing.^1^ However, many patients at risk of TB are unable to produce sputum.^2–4^ The reliance on sputum for diagnosis is a major contributor to the diagnostic gap also in drug-resistant TB (DR-TB). Only 44% of the ∼400,000 annual cases of DR-TB are diagnosed and treated.^5^ Recognising this barrier, the WHO prioritises research on alternative sample types for rapid TB and DR-TB diagnosis.^6,7^ Tongue swabs and sputum-dipped swabs have been identified as promising sample types for diagnosis of pulmonary TB.^8–10^ Although sputum swabs require a sputum specimen, they reduce workflow complexity through rapid automated cell lysis instead of DNA extraction. Wang et al.^11^ recently demonstrated drug resistance profiling from tongue swabs using targeted next-generation sequencing (tNGS). To our knowledge, no study has yet reported DR-TB diagnosis from swabs using a rapid, low-complexity molecular assay – a simple, scalable approach that could transform access to DR-TB testing in high-burden settings.

We conducted a proof of concept study in participants with confirmed rifampicin-resistant TB in the Philippines. Testing was done retrospectively on stored samples from participants recruited to the Rapid Research in Diagnostics Development TB Network DR-TB Cohort (R2D2 DR-TB study).^12^ We included 50 paired swab samples from 25 participants, one tongue swab, and one sputum-dipped swab per participant. The reference standards for *Mycobacterium tuberculosis complex* (MTBC) detection and rifampicin and isoniazid resistance were liquid sputum mycobacterial culture and phenotypic drug susceptibility testing using the BACTEC MGIT 960 system (Becton Dickinson, USA). Participants were a random sample of R2D2 DR-TB participants with rifampicin-resistant TB. We purposefully included 15 participants with confirmed additional resistance to isoniazid. Specimens were collected with regular nylon-flocked swabs (FLOQSwabs® 520CS01, Copan Diagnostics, USA). Tongue swabs were collected from the tongue dorsum by brushing for a minimum of 15 s and were stored dry at −80°C within 24 h of collection.^8,9^ Sputum-dipped swabs were immersed in 400–500 µl of thawed homogenised raw sputum (stored at −80°C) and swirled 10 times for 15 s to cover the swab with sputum. We used the Truelyse device (Molbio Diagnostics, India) for rapid cell lysis of swab samples, followed by polymerase chain reaction (PCR) on the Truelab real-time micro PCR system with MTB Ultima (Lot. EMT07, prototype) for MTBC detection, and MTB-RIF Dx (Lot. 010TX, Cat.No. 601210050) and MTB-INH (Lot. IN002, Cat.No. 601360050) microchips for drug resistance detection (Molbio Diagnostics, India). Thawed swab samples were placed in Truelyse buffer and processed for 90 s in the Truelyse device. After 30–60 s of incubation at room temperature, 6 µl of lysate were loaded onto the MTB Ultima chip and run on Truelab for MTBC detection. If MTBC was detected, 6 µl of lysate each were transferred to the MTB-RIF Dx and MTB-INH chips and run on Truelab. Indeterminate results and errors on any chip were repeated once using the same lysate. To establish proof of concept for detecting MTBC and drug resistance from swab samples, we analysed data focusing on test validity and diagnostic sensitivity among valid test results. For 23 participants, data from prospective sputum-based testing with Truenat MTB Plus, MTB-RIF Dx, and MTB-INH were also available as a comparator.

Truenat MTB Ultima detected MTBC in 20/25 (80%) tongue swabs and 25/25 (100%) sputum-dipped swabs, and no false negative results were observed (see Table). Non-positive-non-negative (NPNN) results for MTBC detection (including indeterminates, invalids, and errors) consisted of four invalids and one error code 3, likely caused by a clogged cartridge due to sample viscosity. Sensitivity for MTBC detection among samples with valid test results was 100% (95% confidence interval [CI]: 83–100) for tongue swabs and 100% (95% CI: 86–100) for sputum-dipped swabs. We proceeded with resistance testing in 20 tongue swabs and 25 sputum-dipped swabs positive for MTBC on MTB Ultima. Final valid results on MTB-RIF Dx could be obtained for 12/20 (60%) tongue swabs and 12/25 (48%) sputum-dipped swabs (Table). 5/8 samples with NPNN results for rifampicin on a tongue swab showed resistance on the sputum swab. 5/13 samples with NPNN results for rifampicin resistance on a sputum swab showed resistance on the tongue swab (5/13 were not tested for resistance with a tongue swab due to NPNN results on the tongue swab MTB Ultima test). MTB-INH yielded valid results for 11/20 (55%) tongue swabs and 16/25 (64%) sputum swabs (Table). 5/9 samples with an NPNN result for isoniazid on a tongue swab showed resistance on the sputum swab. 1/5 samples with an NPNN result for isoniazid resistance on a sputum swab showed resistance on the tongue swab. 29/39 (74%) NPNN results for resistance detection occurred in samples with low bacillary load (cycle-threshold value on MTB Ultima >25). Repeat testing of tongue swabs resolved ∼25% of indeterminate results on MTB-RIF and 29% on MTB-INH of indeterminate results. In sputum-dipped swabs, 7% of indeterminate results on MTB-RIF Dx and 40% of indeterminate results on MTB-INH could be resolved through repeat testing. In comparison, final proportions of indeterminate results in sputum testing ranged from 21% (4/19) on MTB-RIF Dx to 25% (5/20) on MTB-INH. Truenat MTB-RIF-Dx detected resistance in 11/20 tongue swab samples (n = 7 indeterminate results, n = 2 error code 8) and 11/25 sputum swab samples (n = 11 indeterminate results, n = 3 error code 8). Sensitivity for rifampicin resistance detection among samples with valid results was 92% (11/12, 95% CI: 62–100) for both tongue swab and sputum swab samples. Truenat MTB-INH detected resistance in 6/20 tongue swab samples (n = 9 indeterminate results) and 10/25 sputum swab samples (n = 10 indeterminate results, n = 1 error code 8). Sensitivity for isoniazid resistance detection among samples with valid results was 100% (6/6 95% CI: 54–100) for tongue swabs and 91% (10/11, 95% CI: 59–100) for sputum swabs. Sensitivity among samples with valid results of prospective sputum testing with Truenat MTB-RIF-Dx and MTB-INH in this population were 87% (95% CI: 60–98, n = 15) and 90% (95% CI: 56–100, n = 16), respectively.

**Table.** Testing summary for tongue swabs, sputum swabs, and sputum with Molbio Truenat MTB-RIF Dx and MTB-INH.

	Sensitivity (95% CI)	Valid results (N)				NPNN Results (N)		Total (N)
		TP	TN	FP	FN	CT-value^A^ ≥25	CT-value^A^ <25	
Tongue swabs								
MTB Ultima	100% (83–100)	20	0	0	0	4^B^	1^B^	25
MTB-RIF Dx	92% (62–100)	11	–	–	1	7	1	20
MTB-INH	100% (54–100)	6	4	1	0	9	0	20
Sputum swabs								
MTB Ultima	100% (86–100)	25	0	0	0	0	0	25
MTB-RIF Dx	92% (62–100)	11	0	0	1	7	6	25
MTB-INH	91% (59–100)	10	5	0	1	6	3	25
Sputum								
MTB Plus	86% (65–97)	19	0	0	3	0	0	22
MTB-RIF Dx	87% (60–98)	13	–	–	2	3	1	19
MTB-INH	90% (55–100)	9	4	1	1	4	1	20

CI = confidence interval; TP = true positive; TN = true negative; FP = false positive; FN = false negative; NPNN = non-positive-non-negative; CT = cycle-threshold.

A On MTB Ultima or MTB Plus.

B Smear-positivity used as indicator for bacterial load due to absence of MTB Ultima/Plus cycle-threshold value.

Overall, our data provide proof of concept that molecular drug resistance testing from swab samples is feasible, with sensitivities among samples with valid test results comparable to those obtained from sputum testing. High proportions of indeterminate results, particularly in samples with low bacterial load, represent a significant limitation. This observation is consistent with prior reports of drug resistance testing from swabs using tNGS.^11^ Low bacterial load in samples is a known reason for indeterminate results on molecular platforms in various sample types.^13,14^ Increased sampling time might have potential to increase sensitivity and decrease indeterminate results.^15^ Given the urgent need for simplified, non-sputum–based diagnostics in high-risk populations, further research should focus on optimising assay workflows and pre-analytical steps to improve yield from swab samples.
